# Lung Epithelial Cells Induce Both Phenotype Alteration and Senescence in Breast Cancer Cells

**DOI:** 10.1371/journal.pone.0118060

**Published:** 2015-01-30

**Authors:** Masashi Furukawa, Sarah Wheeler, Amanda M. Clark, Alan Wells

**Affiliations:** Department of Pathology, University of Pittsburgh, and Pittsburgh VA Health System, Pittsburgh, Pennsylvania, United States of America; University of Alabama at Birmingham, UNITED STATES

## Abstract

**Purpose:**

The lung is one of the most common sites of breast cancer metastasis. While metastatic seeding is often accompanied by a dormancy-promoting mesenchymal to epithelial reverting transitions (MErT), we aimed to determine whether lung epithelial cells can impart this phenotype on aggressive breast cancer cells.

**Methods:**

Co-culture experiments of normal lung epithelial cell lines (SAEC, NHBE or BEAS-2B) and breast cancer cell lines (MCF-7 or MDA-MB-231) were conducted. Flow cytometry analysis, immunofluorescence staining for E-cadherin or Ki-67 and senescence associated beta-galactosidase assays assessed breast cancer cell outgrowth and phenotype.

**Results:**

Co-culture of the breast cancer cells with the normal lung cells had different effects on the epithelial and mesenchymal carcinoma cells. The epithelial MCF-7 cells were increased in number but still clustered even if in a slightly more mesenchymal-spindle morphology. On the other hand, the mesenchymal MDA-MB-231 cells survived but did not progressively grow out in co-culture. These aggressive carcinoma cells underwent an epithelial shift as indicated by cuboidal morphology and increased E-cadherin. Disruption of E-cadherin expressed in MDA-MB-231 using shRNA prevented this phenotypic reversion in co-culture. Lung cells limited cancer cell growth kinetics as noted by both (1) some of the cells becoming larger and positive for senescence markers/negative for proliferation marker Ki-67, and (2) Ki-67 positive cells significantly decreasing in MDA-MB-231 and MCF-7 cells after co-culture.

**Conclusions:**

Our data indicate that normal lung epithelial cells can drive an epithelial phenotype and suppress the growth kinetics of breast cancer cells coincident with changing their phenotypes.

## Introduction

Breast cancer is the most common cancer in women. In breast cancer patients, the main cause of death is not due to the primary tumor, but from metastases at distant sites. Most of the women with breast cancer receive some form of adjuvant therapy after removal of the primary tumor (if no synchronous extant metastases are noted), although up to one third of them relapse and ultimately die of metastatic breast cancer [1]. Thus, the tumor biology of the micrometastatic niche is critical to reducing the mortality from this dreaded disease.

Curiously, the metastatic process is very inefficient. Many breast cancer cells reach the circulation even from small localized lesions [2]. Yet very few tumor cells in the circulation develop into metastases [3,4]. Experimental studies have long established that only ~0.01% of cancer cells injected into the circulation form detectable metastatic foci [5]. As the ectopic environment is foreign and lacks many of the physiologic trophic factors of the primary tissue this failure to seed and grow should not be surprising [6]. The question remains as to what rare changes occur in the tumor cell to enable survival in the ectopic environment.

During the metastatic seeding of disseminated carcinomas, mesenchymal to epithelial reverting transitions (MErT) are proposed to revert the mesenchymal phenotype that allows for emigration from the primary tumor mass [7,8]. This has been noted in clinical cases where the epithelial marker E-cadherin [9] is upregulated in the metastatic site compared to the primary mass [10,11]. Further, *in vivo* experimental systems have shown this reversion even in highly aggressive breast [11] and prostate [12] cancers when seeding the liver. Thus, MErT is considered to contribute substantially to the colonization of metastatic tumors at the secondary site [8], but this has not been demonstrated for most organs.

Our previous studies have shown that co-culturing of breast cancer cells or prostate cancer cells with hepatocytes drives the E-cadherin re-expression and this phenotypic reversion [11,13]. However, it is not clear that this effect would be universal in target organs, although clinically this MErT alteration is noted in disparate tissues and not just liver [10,13]. As lung is a major site of metastatic seeding, we asked whether the parenchymal cells can impart a MErT. Herein, we report that normal lung epithelial cells (NLC) can drive phenotypic changes in breast cancer cells. Of particularly interest is not just that this coincides with proliferative suppression but a number of these cells are induced into a senescent phenotype.

## Materials and Methods

### Cells and cell culture

Normal lung epithelial cell lines (NLC) SAEC and were purchased from Lonza. BEAS-2B cells were purchased from American Type Culture Collection. SAEC cells were cultured in SAGM medium (Lonza, Anaheim, CA). NHBE and BEAS-2B cells were cultured in BEGM medium (Lonza, Anaheim, CA). The SAEC are derived from smaller airways and alveoli, whereas the NHBE and BEAS-2B cells represent bronchial derivations, with the latter of these being immortalized by SV40 transfection.

The breast cancer cell lines were obtained originally from ATCC. RFP expressing MDA-MB-231 (MDA-MB-231), E-cadherin-MDA-MB-231 (231-Ecad), shRNA-E-cadherin-MDA-MB231 (231-shEcad) and MCF-7 cell lines were transfected as previously described [11]. To maintain selection for RFP positive breast cancer cells, MCF-7 and 231-Ecad cells were cultured with 900 μg/ml G418, 231-shEcad cells were cultured with 900 μg/ml G418 and 5 μg/ml puromycin and MDA-MB-231 cells were cultured with 10 μg/ml puromycin in RPMI-1640 (Life Technologies, Carlsbad, CA) supplemented with 10% FBS until used in the experiments; during the 6 days of experimentation the RFP was stable even in the absence of the selective antibiotics. Human mammary epithelial cells (HMEC) were stained with cell tracker red (Life Technologies).

### Cell co-culture

NLCs were plated at 1x10^5^ cells per well in 6-well plates coated with 1% rat tail collagen in distilled H_2_O (BD Biosciences, San Jose, CA) and allowed to attach overnight. The next day 2 x10^4^ RFP-labeled cancer cells or cell tracker red-labeled HMEC were seeded onto NLCs monolayers. The co-cultured cells were maintained with respective NLCs growth media (BEGM or SAGM medium); no selective pressures where applied during this experimental period. Medium was replenished every other day.

### Imaging

Phase contrast, RFP and β-galactosidase staining images were captured using an Olympus inverted scope and digitally captured using Spot Advanced software NHBE (Diagnostics Instruments, Macomb, Michigan). Confocal images were captured on an Olympus Fluoview 500 scope (Olympus, Center Valley, PA) and captured using Fluoview Viewer.

### Immunofluorescence

Co-culture plates were fixed on day 6 with 4% formaldehyde for 10 minutes followed by permeabilization with 0.1% Triton X-100 for 20 minutes. After blocking with 2% BSA for 1hour, the cells were incubated with primary antibody against E-cadherin (Cat# 610182, BD Biosciences) at 1:100 dilution and Ki-67 (H300) (Cat# sc-15402 Santa Cruz, Dallas, Texas) at 1:50 dilution at 4°C overnight and then with Alexa Fluor 488-conjugated secondary antibodies (Life Technologies) at room temperature for 1 hour. DAPI was applied to stain the nucleus for 5 minutes.

### Senescence-associated β-galactosidase staining

After 120 hours of co-culture, cells were washed with PBS and fixed in fix solution before the staining with X-gal solution according to the manufacturer’s instructions (Senescence Cell Histochemical Staining Kit, Sigma-Aldrich). After 24 hours of incubation at 37°C. DAPI was applied to stain the nucleus [14].

### Flow cytometry

Cell cultures in each well were incubated in 0.5ml trypsin for 15 min until dissociated. 2 ml of PBS with 10% FBS were added to each well, transferred to flow tubes, and pelleted. The media was aspirated, and cell pellets incubated with the reconstituted components of the Alexa Fluor 488 Annexin V/Dead Cell Apoptosis Kit (Life Technologies, Carlsbad, CA) for 15 min. The reaction was terminated by adding 150 μL of the Annexin binding buffer to each tube. Ten μL of CountBright Absolute Counting Beads (Life Technologies, Carlsbad, CA) were added to each tube to compute the number of RFP positive, Annexin V negative breast cancer cells. Cell suspensions were run on a BD FACSCalibur flow cytometer and CellQuest Software (Becton Dickinson, Franklin Lakes, NJ).

### Immunoblotting

A 12% SDS—PAGE gel resolved cell lysates and were subsequently transferred to a PVDF membrane. Membranes were blocked with 5% serum albumin for 1 hour and incubated overnight with primary antibody, E-cadherin primary antibody (24E10) (Cat#3195, Cell Signaling, Danvers, MA), α-catenin primary antibody (25B1) (Cat# ab49105, Abcam), β-catenin primary antibody (Cat# 610153, BD Biosciences), p120-catenin primary antibody(Cat# 610133, BD Biosciences), Connexin 43primary antibody (Cat# 3512, Cell Signaling) or GAPDH primary antibody (Cat# G9545, Sigma). Chemiluminescence was detected on film following incubation with peroxidase-conjugated secondary antibodies.

## Results

### Normal lung epithelial cells support survival and clustering of breast cancer cells

Our previous studies have shown that primary human hepatic parenchymal cells drive an epithelial phenotype and dormancy in the liver [11,15], whereas non-parenchymal cells or endothelial cells in tissue culture confer a mesenchymal phenotype and growth advantage to breast cancer cells [16]. The latter was likely due to the non-parenchymal cells being stressed on stiff culture surfaces. We investigated which phenotype NLC would promote in breast cancer cells. That NLC could alter cancer cells was suggested by alteration of prostate cancer cell phenotype by lung cells [17].

Co-culture of the breast cancer cells and NLCs was performed by first seeding the NLCs in their respective medium overnight to allow attachment. Then, the breast cancer cells were seeded. The breast cancer cells were seeded under four culture conditions: 1) co-cultured with SAEC cells in SAGM medium, 2) co-cultured with BEAS-2B cells in BEGM medium, 3) mono-cultured breast cancer cells in SAGM medium, 4) mono-cultured breast cancer cells in their normal growth medium (RPMI with 10% serum). The cells were evaluated by live imaging over the time period, with terminal experiments for flow cytometric enumerations.

**Fig 1:**
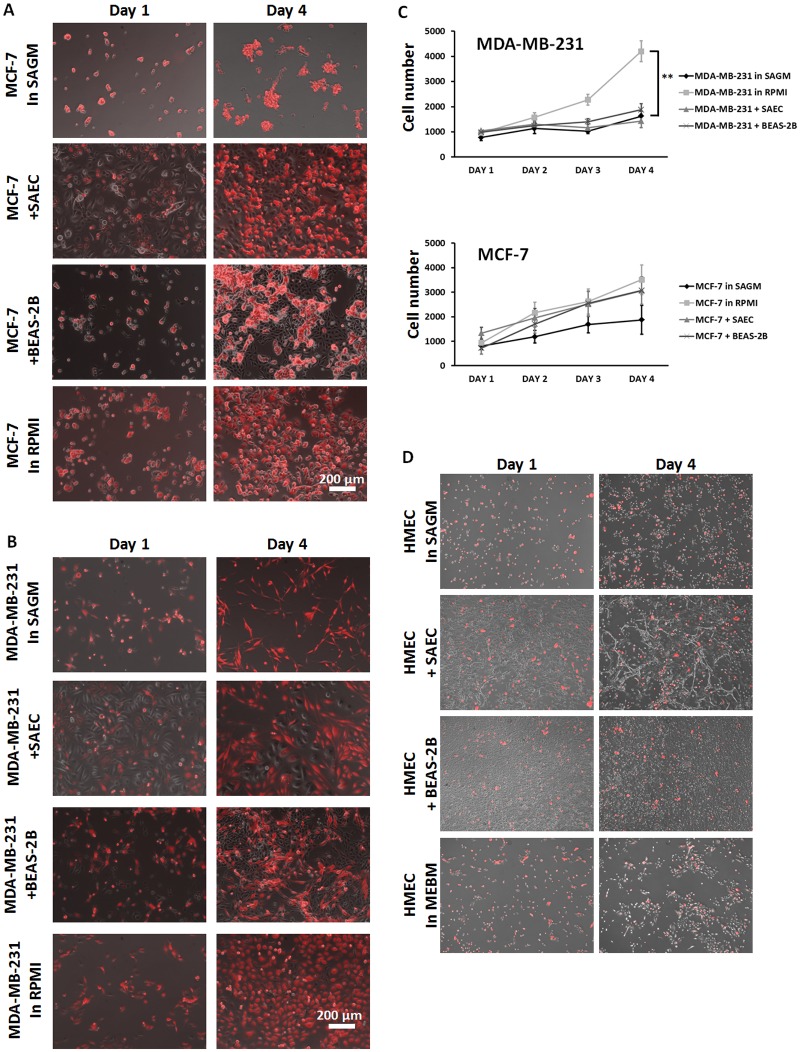
Co-culture of normal lung epithelial cells with breast cancer cells lines. Phase contrast imaging with a fluorescent overlay ((A) Red MCF-7 cells, (B) Red MDA-MD-231 cells, (D) HMEC) as representative images on Day 1 versus Day 4. Scale bar 200 μm. (C) Flow cytometry quantified Annexin V negative, RFP-positive breast cancer cells in 4 different conditions: breast cancer cells in SAGM or RPMI (normal growth) medium, breast cancer cells co-cultured with SAEC or BEAS-2B. Day 0 the NLCs were plated on 12 well plates and allowed to adhere for 3 h. breast cancer cells were then inoculated into the cultures. Days 1–4 represent consecutive 24 h harvesting post breast cancer cells seeding. Immunofluorescent images shown are representative of three separate experiments. In (C) shown is mean ± s.e.m. of three experiments in triplicate. **p value < 0.01 using ANOVA comparing the MDA-MB-231 in RPMI against all other groups.

MCF-7 cells survived in tight clusters in SAGM medium (Fig. 1A top), but MDA-MB-231 cells remained dispersed (Fig. 1B). The MCF-7 cells expanded in co-culture with SAEC or BEAS-2B as observed by microscopy and flow cytometry, all the while staying clustered, even more so than the usual growth in RPMI supplemented with serum. The MDA-MB-231 cells formed clusters in co-culture rather than growing as dispersed mesenchymal cells in monoculture, and co-cultured cells appeared not to expand in number compared to the breast cancer cells grown in full media (RPMI).

More precise enumeration of cells by flow cytometric analyses demonstrated enhanced growth over media alone for MCF-7 in co-culture (Fig. 1C bottom). Both small and large airway epithelial cells drove MCF-7 growth compared to the unsupplemented minimal media (SAGM) that supported survival but not increase in cell number. For the MDA-MB-231 cells, co-culture did not increase growth over the minimal media (Fig. 1C top). The question arises as to whether the NLCs would drive proliferation of non-transformed cells. We did not find this when we co-cultured normal mammary epithelial cells (HMEC) with or without NLCs (Fig. 1D).

### NLCs decrease Ki-67 positivity in breast cancer cells

**Fig 2:**
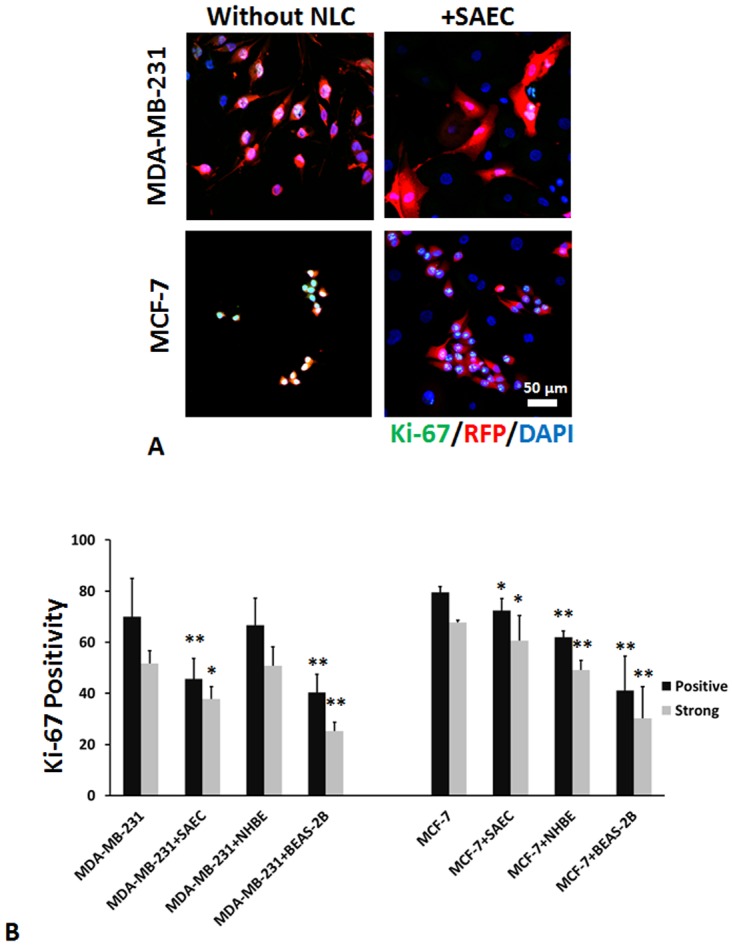
Proliferative fraction of breast cancer cells in co-culture. (A) Representative immunofluorescence images of Ki67 staining of MDA-MB-231 and MCF-7 cells in monoculture versus co-culture with SAEC on day 6 after seeding. Scale bar 50 μm, shown are representative of three experiments using confocal imaging. (B) Percent Ki-67 positivity of just the MDA-MB-231 or MCF-7 with or without co-culture. Shown are mean ± s.e.m. *p value < 0.05, compared to the controls using Chi-squared comparisons to distinguish differences in frequencies in independent populations.

To investigate whether the proliferative fraction of the breast cancer cells was altered, the fraction of breast cancer cells in mitogenic cycle, as noted by expression of Ki-67, was determined. Fluorescent images depict MDA-MB-231 and MCF-7 cells in monoculture versus co-culture with SAEC (Fig. 2A). After co-culture, the fraction of cells staining for Ki-67 was significantly decreased though by a small fraction, with the exception of MDA-MB-231 cells co-cultured with NHBE cells (Fig. 2B). Interestingly, despite the seemingly high number MCF-7 cells in co-culture, the proliferative fraction was reduced, even by BEAS-2B cells. While the actual decrement is small, it should be noted that only a fraction of tumor cells are reverted by hepatocytes in 2D culture [11], or undergo MErT in 3D microphysiologic systems [18,19].

### Normal epithelial lung cells modulate MDA-MB-231 phenotype

**Fig 3:**
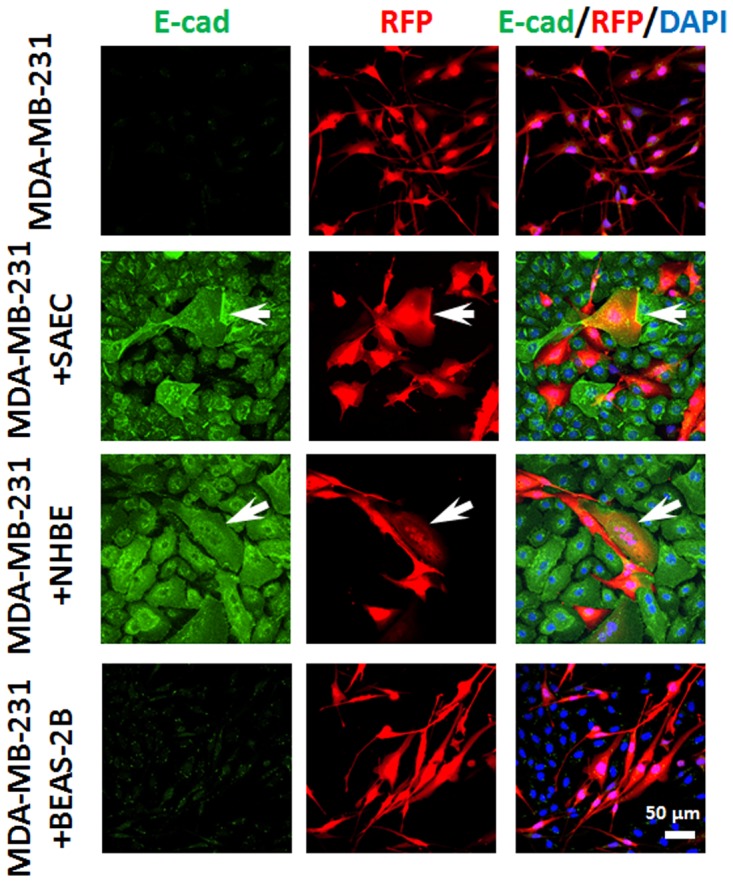
E-cadherin expression in breast cancer co-cultures. Representative immunofluorescence images: MDA-MB-231 co-culture with normal lung epithelial cells. In co-culture with SAEC or NHBE cells, MDA-MB-231 cells changed morphology with some becoming E-cadherin positive (white arrows). In co-culture with BEAS-2B, there was no similar change. Scale bar 50 μm. Images are from 5 days of culture. Shown are representative images from three independent experiments using confocal imaging.

**Fig 4:**
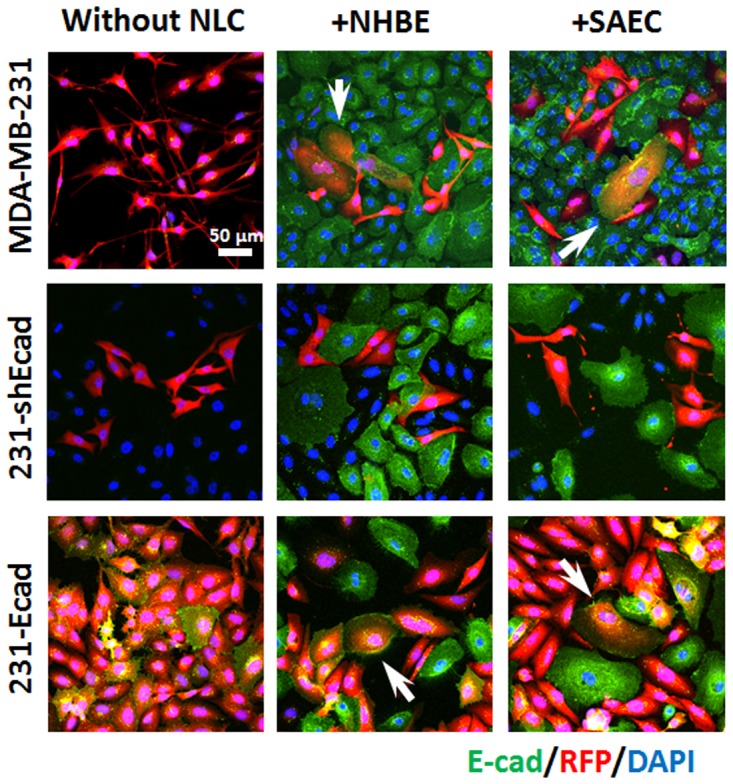
Breast cancer cell morphology in co-culture dependent on E-cadherin expression. Representative immunofluorescence images of MDA-MB-231, 231-shEcad, 231-Ecad and co-cultured with NHBE or SAEC. MDA-MB-231 and 231-Ecad present as cuboidal, bigger cells expressing E-cadherin; 231-shEcad cells did not show these changes. Scale bar 50 μm. Images are from 5 days of culture. Shown are representative images from two independent experiments using confocal imaging.

We have previously published that the MDA-MB-231 cells in hepatocyte co-cultures undergo MErT characterized by the re-expression of E-cadherin and a decreased proliferative rate [11]. In the MDA-MB-231 cells and NHBE or SAEC co-cultures, a sub-population of the MDA-MB-231 cells presented an epithelial shift as indicated by cuboidal morphology, increased size, and E-cadherin positivity. These changes were observed in 8.9±2.6% of MDA-MB-231 cells co-cultured with SAEC and 6.6±0.9% of MDA-MB-231 cells co-cultured with NHBE; but these changes were not noted in co-culture with BEAS-2B (Fig. 3); again, the difference with these cells is being immortalized with SV40 and being of the large airway epithelium, though the NHBE large epithelial cells induced changes similar to the small airway cells. The phenotypic changes noted in the MDA-MB-231 cells were also seen in the cells in which E-cadherin expression is forced (231-Ecad cells). These changes were observed in 4.8±1.8% of 231-Ecad cells co-cultured with SAEC and 4.4±1.3% of 231-Ecad cells co-cultured with NHBE. Abrogation of E-cadherin expression by antisense RNA prevented this phenotypic and morphologic shift (231-shEcad cells) demonstrating E-cadherin dependency (Fig. 4).

### SAEC confers a cellular senescence to MDA-MB-231 cells

**Fig 5:**
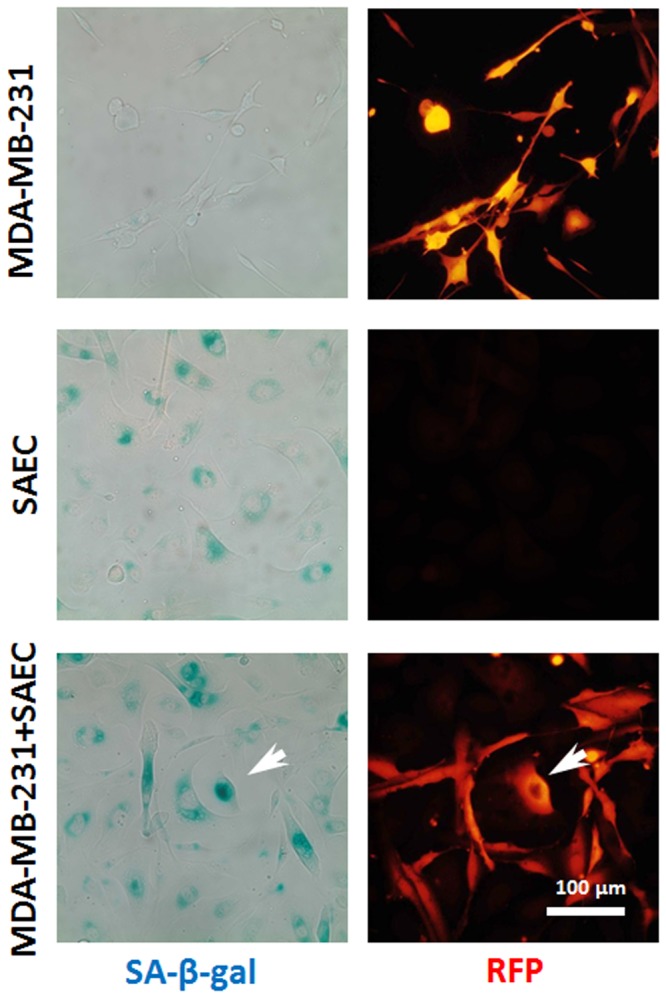
Senescence of breast cancer cells in co-culture. Representative immunofluorescent images from three independent experiments of MDA-MB-231, SAEC and MDA-MB-231 co-cultured with SAEC, for 5 days and stained for induction of senescence. Blue stains reveal increased SA-β-gal activity indicative of cellular senescence. MDA-MB-231 cells are RFP labeled. Scale bar 100 μm. White arrows—morphology changed MDA-MB-231 cells positive for SA-β-gal.

To further investigate the question of suppressing tumor outgrowth in the metastatic niche we examined whether these cells underwent senescence [20]. In addition, we had noted enlarged MDA-MB-231 cells in the co-cultures with SAEC and NHBE but not BEAS-2B cells which suggested senescence. A subset of these cells stained with the senescence associated marker β-galactosidase (SA-β-gal) (Fig. 5). These changes were observed in 3.9±1.5% of MDA-MB-231 cells co-cultured with SAEC. Interestingly, we did not find SA-β-gal staining in the MCF-7 cells (data not shown).

### Discussion

We found that normal lung epithelial cells modulated breast cancer cell behaviors. After co-cultured with NLCs, MDA-MB-231 cells presented a more epithelial phenotype with some of the aggressive cancer cells reverting and expressing E-cadherin. Thus for the first time it was shown that lung epithelial cells could impact cancer cell phenotype similarly to previous findings with hepatocytes [11,15]. New to this study, we found that a small subset of the MDA-MB-231 cells now appeared to be senescent being larger and expressing the senescence-associated beta-galactosidase and lacking Ki67 positivity. In conjunction, the NLCs suppressed the proliferative rate of MDA-MB-231. On the other hand, the NLCs also alter the epithelial MCF-7 breast cancer cells to converge with the MDA-MB-231 cells in terms of establishment and morphology. The key issue is that these data demonstrate for the first time that lung epithelial cells can impart phenotypic changes to cancer cells as was shown earlier for hepatocytes [11,12] demonstrating the generalizability of this cell-cell communication.

These findings are also consistent with reports of phenotypic reversion of these and other tumor breast tumor cells when introduced back into the orthotopic environment of a mammary fat pad [21,22,23]. In these *in vivo* studies and in those that report epithelial phenotype in human metastatic breast cancer specimens [10,13,24], the contribution of the different cell types and regenerative micro-environment cannot be individually discerned. This distinction is important as we have found that activated stromal cells can drive a mesenchymal transition in the epithelial MCF-7 breast carcinoma cells [16], whereas stromal cells integrated within a non-inflamed liver microphysiologic system contribute to the suppression of malignancy and a shift towards an epithelial phenotype [19]. The effects of the local or metastatic microenvironment appear to depend on the underlying state of the tissue at the time of tumor cell introduction.

The situation in 2D culture does not fully reflect the tissue microenvironment in a tissue due to many factors. One main issue is the stiffness of the supporting substratum. Pathological, and in tissue culture suprapathological, stiffness is well known to lead to a mesenchymal transition of even non-neoplastic breast epithelial cells [25,26]. Still this does not account for the transformation seen in MCF-7 cells to a more spindle-like morphology as these cells form epithelial clusters on the same substratum when in isolation. However, this stiffness also imparts an ‘inflammation’ or stressed phenotype to the normal cells that can lead to secreted factors that drive breast cancer cell progression [16]. While such studies lie beyond the scope of this initial missive, the overall net behavior of these NLCs on both the epithelial MCF-7 and mesenchymal MDA-MB-231 cells is to decrease the proliferative fraction (as noted by Ki67) and impart a (pre-)dormant phenotype that is noted in both 3D culture models [18,27] and in patients [13].

Of note in our findings is that the BEAS-2B cells did not drive the epithelial transition or senescence of the MDA-MB-231 cells, unlike the SAEC and NHBE cell co-cultures. Further, these cells grow faster in culture and have a different morphology from the other two normal lung epithelial cells, and stain weakly for E-cadherin by immunofluorescence (S1 Fig.), though an antibody to a different epitope provides strong immunoblotting (S2 Fig.). BEAS-2B cells differ in two key ways being SV40 immortalized and derived from the large airway epithelium, though the NHBE cells also represent large airway derivations without the confounding immortalization. As most breast cancer metastases originate in the small airways, large airway epithelial cells may not provide the key signals for the epithelial transition. Despite the immortalization of the BEAS-2B cells, the BEAS-2B cells do suppress the proliferative index of both breast cancer cell lines.

Our data suggest NLCs in the lung metastatic microenvironment may contribute to the metastatic outcome of disseminated breast cancer cells. Similar to hepatocytes, these normal cells may promote the survival, seeding and even dormancy of aggressive mesenchymal carcinoma cells. The NLCs also provide progressive signaling when stressed accounting for the spindle-like morphology and slightly increased growth rates of the epithelial carcinoma cells, such as MCF-7. In this latter manner, insults to the small airway may provoke an outgrowth of dormant breast cancer cells. This speculation, which is the subject of planned future investigations, will need to be tested in developed organ culture systems with putative irritants and stressors, both biological and environmental, and in animal models.

## Supporting Information

S1 Fig Morphology of normal lung epithelial cells. Representative phase contrast (left column) and confocal immunofluorescence of E-cadherin (right column) images of the normal lung cell lines used herein. Black scale bar 200 μm. White scale bars 50 μm.
(TIF)

S2 Fig Expression of epithelial markers in the cell lines used herein. Representative immunoblots for E-cadherin and other proteins indicative of the epithelial phenotype in breast cancer cells and normal lung epithelial cells. One of more than three experiments is shown.
(TIF)
