# Supplementary figures and images for: Lung Epithelial Cells Induce Both Phenotype Alteration and Senescence in Breast Cancer Cells
Source: PLoS One. 2015 Jan 30;10(1):e0118060. doi: 10.1371/journal.pone.0118060 (PMC4311980; doi:10.1371/journal.pone.0118060)

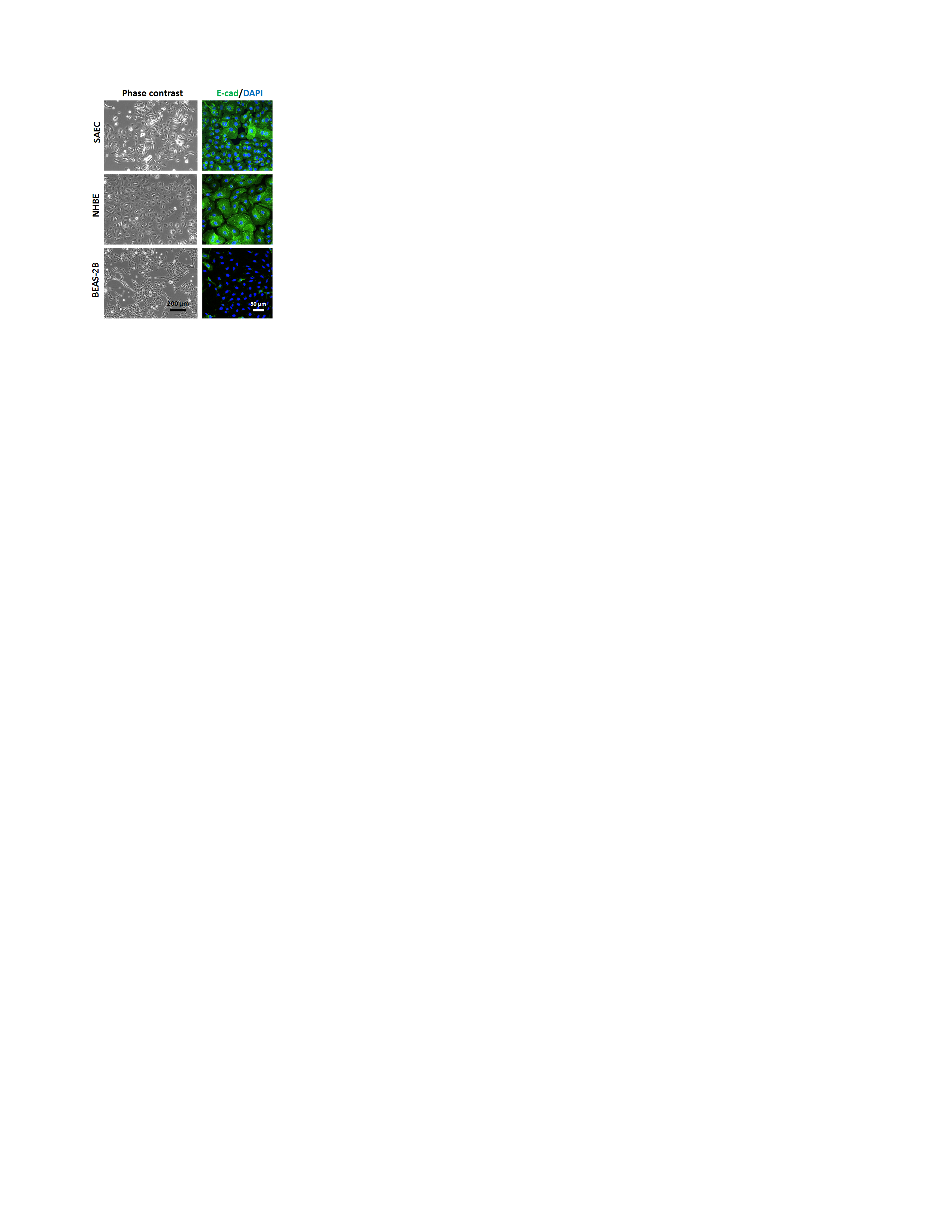

Supplement: S1 Fig — Representative phase contrast (left column) and confocal immunofluorescence of E-cadherin (right column) images of the normal lung cell lines used herein. Black scale bar 200 μm. White scale bars 50 μm. (TIF) [file pone.0118060.s001.tif]

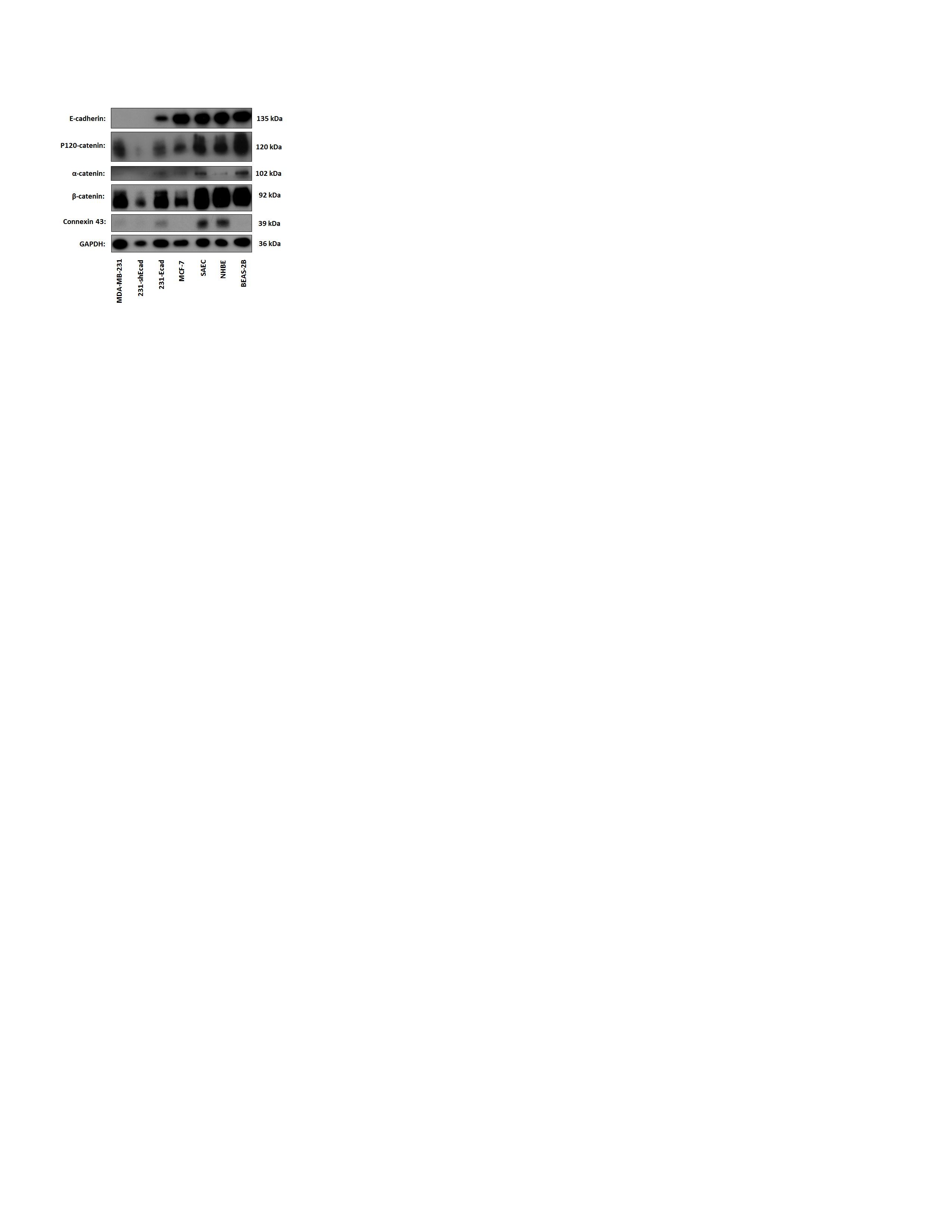

Supplement: S2 Fig — Representative immunoblots for E-cadherin and other proteins indicative of the epithelial phenotype in breast cancer cells and normal lung epithelial cells. One of more than three experiments is shown. (TIF) [file pone.0118060.s002.tif]
